# Lysosomal acidification dysfunction in microglia: an emerging pathogenic mechanism of neuroinflammation and neurodegeneration

**DOI:** 10.1186/s12974-023-02866-y

**Published:** 2023-08-05

**Authors:** Joseph D. Quick, Cristian Silva, Jia Hui Wong, Kah Leong Lim, Richard Reynolds, Anna M. Barron, Jialiu Zeng, Chih Hung Lo

**Affiliations:** 1 Department of Integrative Biology and Physiology, Medical School, University of Minnesota, Minneapolis, MN USA; 2 Faculty of Graduate Studies, University of Kelaniya, Kelaniya, Sri Lanka; 3 Lee Kong Chian School of Medicine, Nanyang Technological University, Singapore, Singapore; 4 Department of Brain Sciences, Faculty of Medicine, Imperial College London, London, UK

**Keywords:** Lysosomal acidification, Autophagy, Phagocytosis, Cytokines, Toxic protein aggregates, Neuroinflammation, Neurodegenerative diseases, Acidic nanoparticles

## Abstract

Microglia are the resident innate immune cells in the brain with a major role in orchestrating immune responses. They also provide a frontline of host defense in the central nervous system (CNS) through their active phagocytic capability. Being a professional phagocyte, microglia participate in phagocytic and autophagic clearance of cellular waste and debris as well as toxic protein aggregates, which relies on optimal lysosomal acidification and function. Defective microglial lysosomal acidification leads to impaired phagocytic and autophagic functions which result in the perpetuation of neuroinflammation and progression of neurodegeneration. Reacidification of impaired lysosomes in microglia has been shown to reverse neurodegenerative pathology in Alzheimer’s disease. In this review, we summarize key factors and mechanisms contributing to lysosomal acidification impairment and the associated phagocytic and autophagic dysfunction in microglia, and how these defects contribute to neuroinflammation and neurodegeneration. We further discuss techniques to monitor lysosomal pH and therapeutic agents that can reacidify impaired lysosomes in microglia under disease conditions. Finally, we propose future directions to investigate the role of microglial lysosomal acidification in lysosome–mitochondria crosstalk and in neuron–glia interaction for more comprehensive understanding of its broader CNS physiological and pathological implications.

## Background

Microglia are a type of glial cells that represent 5–15% of the adult brain cells [1]. Being professional phagocytes, microglia maintain brain homeostasis under physiological conditions by phagocytosing neuronal synapses, apoptotic cells, and cellular debris, as well as supporting neuronal development and survival by releasing trophic factors [2]. Microglia also form the largest population of resident immune cells which adopt a macrophage-like role in the brain, making them indispensable in cultivating and maintaining the architecture of the central nervous system (CNS) [3, 4]. Under stimulated or diseased conditions, microglia become activated which result in changes in morphology, cytokine production and secretion, and phagocytic capacity [5]. In general, microglia activation and the associated increased cytokine release are intended to be neuroprotective, although chronic microglia activation and excessive neuroinflammatory responses can be detrimental to the brain, leading to the pathogenesis of neurodegenerative diseases such as Alzheimer’s disease (AD) [6]. Microglia can display a spectrum of heterogenous states including homeostatic microglia and those that respond to stimuli with distinct profiles of cytokine expression [2, 7–9]. The specific microglial phenotype invoked is largely a result of environmental signals, not only from neurons and other glial cells, but also from immune cells within the perivascular spaces [2, 10].

**Fig. 1:**
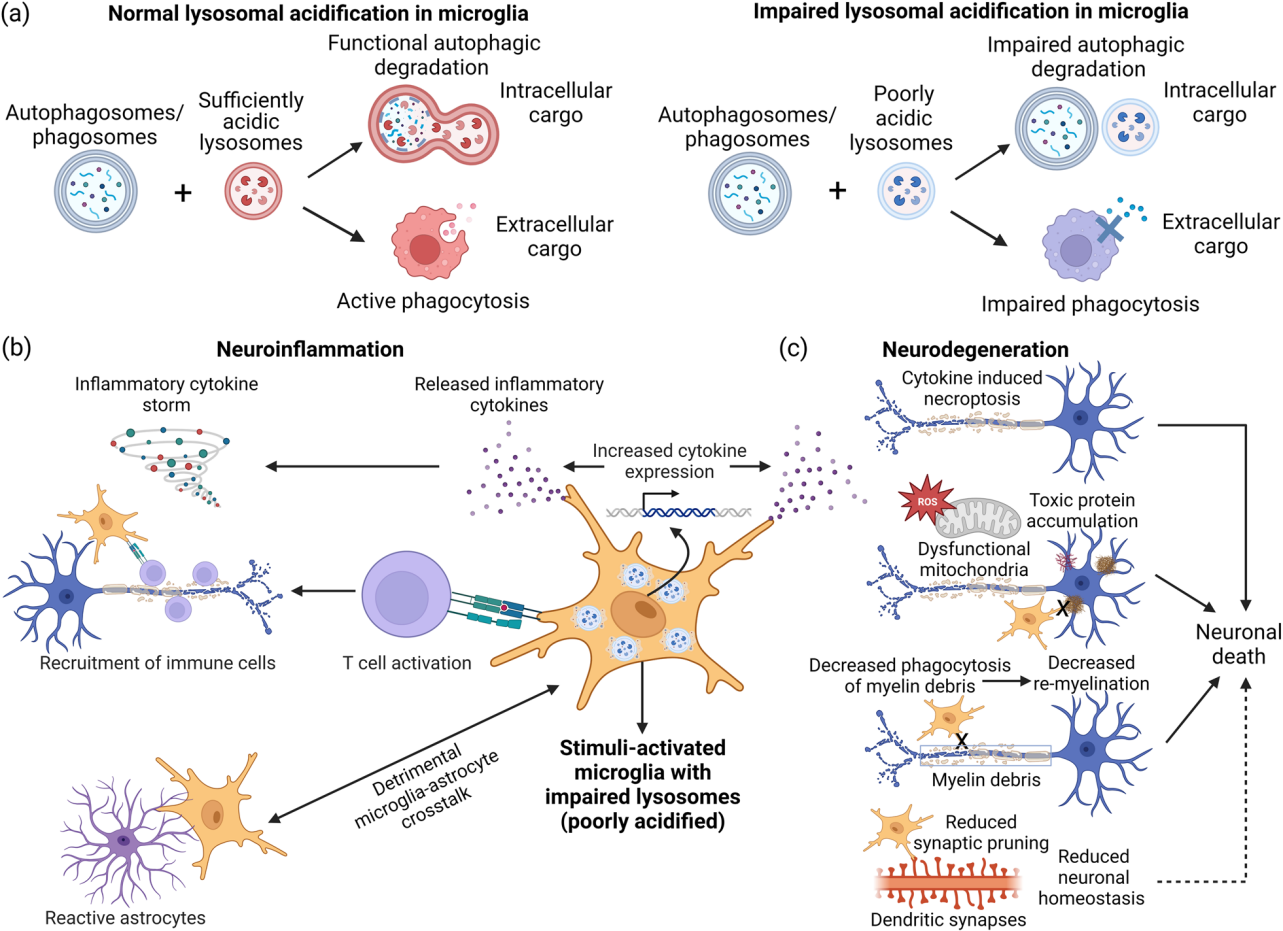
Implications of microglial lysosomal acidification dysfunction in neuroinflammation and neurodegeneration. **a** In healthy microglia, lysosomes with sufficiently acidic lumen can fuse with autophagosomes or phagosomes to form functional autolysosomes for efficient degradation of both intracellular and extracellular cargoes (left). Under stimulated or diseased conditions, lysosomes with poorly acidic lumen (impaired lysosomal acidification) will have inefficient fusion with autophagosomes or phagosomes, or even no fusion, leading to reduced phagocytic and autophagic functions (right). **b** In the context of neuroinflammation, stimuli-activated microglia with impaired lysosomal acidification express and secrete more cytokines to perpetuate neuroinflammation. Through releasing more inflammatory cytokines, these dysfunctional microglia recruit and activate immune cells and participate in detrimental crosstalk with astrocytes to propagate inflammatory response. **c** In the context of neurodegeneration, increased inflammatory cytokine secretion by dysfunctional microglia with lysosomal acidification defect contributes to neuronal death via mechanisms such as necroptosis. In addition, these impaired microglia have reduced phagocytic and autophagic capabilities in the clearance of toxic protein aggregates, damaged organelles such as mitochondria, and myelin debris, as well as in synaptic pruning, leading to eventual neuronal death and neurodegeneration. The figure was created with BioRender.com

The development of microglia and their responsiveness to stimuli or stress have been reported to be dependent on a functional lysosomal regulatory circuit [11]. Additionally, microglial phagocytosis and autophagy require proper lysosomal acidification and functions to complete the cellular degradation and recycling system [11–13]. Lysosomal acidification is regulated by the membrane-bound vacuolar (H +)-ATPase (V-ATPase) and ion channels [14, 15]. Being the final step of the clearance process, the extent of lysosomal acidification and its enzyme activities play critical roles in substrate metabolism and degradation of endogenous cellular waste (intracellular cargo) and ingested proteins (extracellular cargo) through autophagy and phagocytosis, respectively [12, 16, 17] (Fig. 1a), thus controlling microglial function and determining the degree of their activation under stimulated or diseased conditions.

Defective lysosomal acidification in the microglia contributes to neuroinflammation and neurodegeneration through a variety of deleterious cellular pathways. Stimuli-activated microglia with defective lysosomal acidification produce and release more inflammatory cytokines [18, 19], recruit and activate immune cells [12, 20], and participate in detrimental crosstalk with astrocytes to exacerbate neuroinflammation [21] (Fig. 1b). On the other hand, dysfunctional microglia with lysosomal acidification impairment and the associated lysosomal membrane permeabilization (LMP) increase inflammatory cytokine secretion and induce neuronal death via mechanisms such as necroptosis [22, 23]. In addition, these impaired microglia have reduced phagocytic and autophagic capabilities in the clearance of damaged organelles such as mitochondria [24, 25], toxic protein aggregates [26], and myelin debris [27], as well as in synaptic pruning [3], leading to eventual neuronal death and neurodegeneration (Fig. 1c). Restoration of microglial autophagy and phagocytosis functions have been shown to be critical for mitigating neuroinflammation [28] and neurodegeneration [12], suggesting the need to modulate these processes, including the modulation of microglial lysosomal acidification. However, the exact mechanisms inducing lysosomal acidification impairment and the consequences of lysosomal pH modulation in microglial function and activation remain unclear and need to be investigated [29].

In this review, we summarize key factors and mechanisms that contribute to lysosomal acidification dysregulation in microglia, including presenilin modification, cytokine and inflammatory stimulation, metabolite and lipid dysregulation, adenosine triphosphate (ATP) level and purinergic receptors signaling, exposure to toxic protein aggregates, lysosome physiology and cathepsin activity, with relevance to the pathogenesis of neuroinflammation and neurodegeneration. In addition, we discuss techniques used for lysosomal pH monitoring, as well as therapeutic agents that have been demonstrated to be effective in restoring lysosomal and autophagic function in CNS cells including microglia. We conclude by proposing future investigations to understand the role of microglial lysosomal acidification in lysosome–mitochondria crosstalk and in the interactions among microglia, astrocytes, and neurons to better elucidate neurodegenerative mechanisms and derive neuroprotective strategies.

## Role of presenilins in autolysosomal function of microglia

Presenilin 1 (*PSEN1*, PS1) and presenilin 2 (*PSEN2*, PS2) are proteins responsible for the enzymatic activity of γ-secretase, a multi-subunit intramembrane protease complex that plays a significant role in the cleavage of amyloid precursor protein (APP) to generate beta-amyloid (Aβ) [30]. PS1 mutations are commonly associated with early-onset familial AD (FAD) [31]. While PS1 is broadly expressed in the CNS, it is highly expressed in microglia within certain regions of the brain such as the cortex [32]. PS1 plays important roles in modulating microglial activation and inflammatory cytokine release [33]. For instance, in BV2 mouse microglial cell lines, γ‐secretase inhibitors modulated pro‐inflammatory cytokine expression upon lipopolysaccharide (LPS) treatment [34]. Furthermore, PS1 plays a critical role in modulating lysosomal acidification in microglia [32], in addition to its known role in neurons [35], and its malfunction contributes to neuroinflammation and neurodegeneration [33, 36–39].

**Fig. 2:**
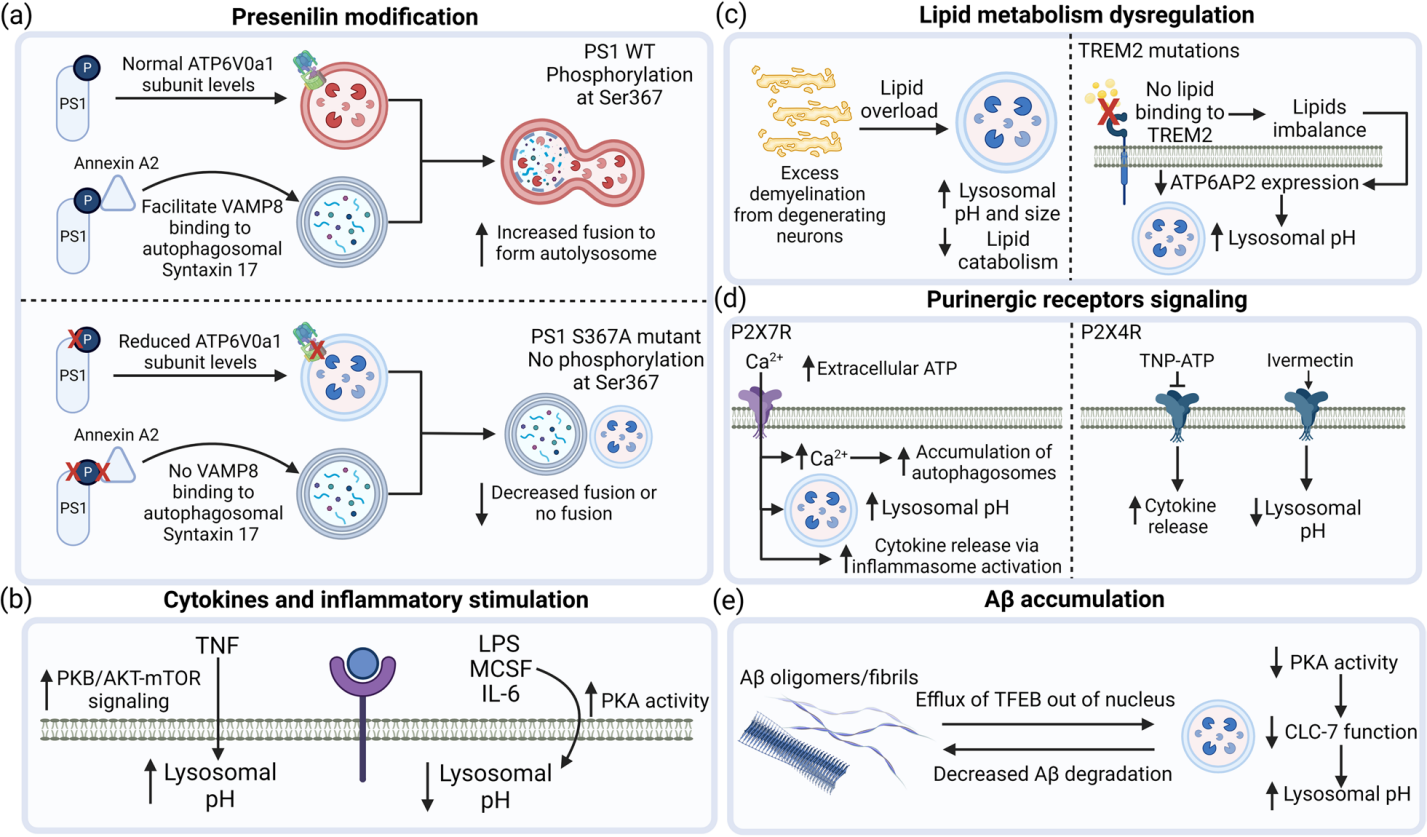
Factors affecting microglial lysosomal acidification and associated phagocytic and autophagic function. **a** Presenilin-1 (PS1) deficient in phosphorylation at Ser367 (S367) leads to reduced ATP6V0a1 levels, impairing lysosomal acidification. Furthermore, PS1 S367A reduces binding to Annexin A2 and decreases VAMP8 binding to autophagosomal Syntaxin 17, thereby preventing autophagosome-lysosome fusion, leading to autophagic inhibition. **b** Cytokine stimulations induce differential effects of lysosomal acidification alterations in microglia. **c** Lipids such as myelin debris and TREM2 mediated lipid accumulation impair microglial lysosomal acidification through different mechanisms and decrease lipid catabolic activity. **d** Purinergic receptors signaling regulate lysosomal acidification. Extracellular ATP activate P2X7R, leading to influx of Ca^2+^, accumulation of autophagosomes, elevation of lysosomal pH as well as increased cytokine release via inflammasome. On the other hand, inhibition of P2X4R by TNP-ATP results in increased cytokine release, while activation of P2X4R by ivermectin improves lysosomal acidification and promotes microglial function. **e** Accumulation of Aβ protein has been shown to lead to defective lysosomal acidification, potentially due to efflux of TFEB out of nucleus. Similarly, a decrease in PKA activity along with decreased ClC-7 chloride transporter function leads to lysosomal pH elevation, resulting in reduced degradation of Aβ, further driving neurodegeneration. The figure was created with BioRender.com

PS1 can be phosphorylated at several sites and the extent of PS1 phosphorylation can affect its function in different ways, including the regulation of cell growth and differentiation [38]. Importantly, phosphorylation of PS1 on its third intracellular loop at Ser367 increases microglial lysosomal acidification and autophagic degradation of APP β-C-terminal fragment (APP-βCTF), the precursor of Aβ [38]. Using a double knock-in mouse model (*PS1*^*S367A/S367A*^), it was shown that there is significant accumulation in levels of APP-βCTF, and inhibition of autophagic flux, together with lysosomal acidification defects in microglia [32]. PS1 Ser367 phosphorylation exerts its effect on microglial lysosomal acidification and associated autophagic function in two ways (Fig. 2a): (1) lack of PS1 phosphorylation at Ser367 reduce lysosomal V-ATPase subunit ATP6V0a1 levels, contributing to impaired assembly of the lysosomal V-ATPase, thereby leading to lysosomal pH elevation [32]; and (2) PS1 phosphorylated at Ser367 specifically binds Annexin A2, which facilitates the binding of lysosomal soluble *N*-ethylmaleimide-sensitive factor attachment protein receptor (SNARE) Vamp8 to the autophagosomal SNARE Syntaxin 17 to modulate the fusion of autophagosomes with lysosomes [38]. Interestingly, PS1 knockout microglia exhibit normal lysosomal acidification with no change in ATP6V0a1 levels [32], indicating that the change in microglial lysosomal pH is not due to loss of PS1 function, which has been shown to be the cause of lysosomal acidification dysfunction in neurons [35, 37], but specifically due to phosphorylation of PS1 at Ser367.

Microglial expression of PS2 with N141I mutation resulted in impaired *γ*-secretase activity as well as increased inflammatory cytokine release. Furthermore, in mice with PS2 N141I mutation, in the absence of inflammatory stimuli, there is enhanced microglial *IL-6* and *TREM2* expression in brain as well as reduced microglial branch number and length, indicating microglia activation [40]. In the presence of inflammatory insult LPS, a higher inflammatory gene expression of *IL-6* and *CXCL2*, as well as higher nuclear factor kappa B (NF-κB) transcriptional activity is observed in PS2 N141I mouse brain [40]. Lastly, PS1 and PS2 mutants and knockouts have been found to impair microglial phagocytosis of Aβ through either reducing its cytoskeletal structure (PS1) [41] or reduce mitochondrial function (PS2) [42]. Taking into consideration that presenilins play important roles in regulating lysosomal activity and microglial function, a clearer understanding of each of the contributions of PS1 and PS2 as well as their potential interplay, should be further elucidated to clarify the molecular mechanisms regulated by presenilins in microglia.

## Differential effects of cytokines and inflammatory stimulation on microglial lysosomal acidification

In the brain, cytokines and inflammatory insults have been shown to regulate immune activation and cell death [29]. As the expression and production of inflammatory cytokines in microglia is much higher than other cell types, microglia plays a major role in driving age-related neuroinflammation [3, 4]. Microglia with a ramified morphology are homeostatic and have little or no phagocytic or inflammatory activity, but are capable of transforming into fully phagocytic and inflammatory activated phenotypes with an amoeboid morphology, upon exposure to different cytokine stimulations [43]. Under different disease states, microglia respond differently to inflammatory stimuli and adopt different cytokine gene profiles [44–50].

Inflammatory stimulations have been shown to lead to microglial lysosomal acidification impairments. However, the contribution of each inflammatory stimulus towards lysosomal acidification remains to be elucidated. For example, while TNF impairs lysosomal acidification in BV2 microglial cells [18], IL-6 promotes lysosomal acidification in primary mouse microglia [51]. LPS exposure in primary mouse microglia lowers lysosomal pH [51], and reduces Aβ burden in APP transgenic mice, potentially through increased microglial phagocytic function [52]. However, in another study it has been shown that while LPS increased lysosomal acidification in N9 mouse microglial cells, autophagic flux is interrupted [53]. This is potentially due to LPS reducing the expression level of VPS34 protein, which is involved in autophagosome biogenesis, leading to autophagosome formation impairment and defective autophagic degradation [53]. Importantly, when conditioned media from activated microglia that has been treated with LPS are treated to neurons, it further induced autophagy inhibition in neurons, leading to neurodegeneration [54]. Hence, different pro-inflammatory cytokines can exhibit differential effects on microglial lysosomal acidification and its associated functions (Fig. 2b).

TNF triggers the phosphorylation of protein kinase B (PKB) or AKT and mTOR signaling, leading to impaired autophagic flux associated with lysosomal acidification dysfunction in mouse primary microglia and BV2 microglial cell lines [18]. In the same study, TNF induces the mouse primary microglia to increase the gene expression of pro-inflammatory genes *iNOS*, *NO*, *IL-1β*, and *IL-6* and a decrease in anti-inflammatory genes including *Arginase1, Ym1/2, and IL-10*. Furthermore, MES23.5 neuronal cells exposed to TNF stimulated microglia conditioned medium exhibit increased cleaved caspase-3 level, indicative of neurotoxicity [18]. This conditioned medium-induced toxicity may derive from an accumulation of pro-inflammatory cytokines or other neurotoxic factors, although no exact characterization of the species was done in this study. Treatment with mTOR inhibitors rapamycin and resveratrol and serum deprivation reversed the deleterious effects via improvement of autophagic function in microglia. Interestingly, the same study also found that TNF promoted transcription factor EB (TFEB) nuclear translocation and increased lysosomal proteins, which is suggested to be due to a compensatory effect of the lysosome to expand in its size to accommodate the accumulated protein loads [18].

Macrophage colony-stimulating factor (MCSF), which has the ability to activate the pro-inflammatory character of microglia, has also been shown to promote lysosomal acidification to increase the degradation of fibrillar Aβ in primary mouse microglia [51]. Specifically, MCSF addition increases ClC-7 and osteoporosis-associated transmembrane protein 1 (OSTM1) transcription and interactions in primary mouse microglia, allowing for proper ClC-7 chloride channel trafficking and complete lysosomal acidification [55]. Treatment with either a protein kinase A (PKA) inhibitor or a chloride channel inhibitor partially reversed the acidifying effects of MCSF in primary mouse microglia, indicating that MCSF is acting through PKA-mediated chloride channel phosphorylation [51]. In AD mice, MCSF treatment decreased Aβ plaque density [51], and prevented cognitive decline [56], due to the increased number of microglia as a result of increased infiltration of bone marrow donor cells in response to systemic MCSF treatment and derivation into microglia. As different cytokines have opposing effects on modulating microglial lysosomal acidification and function, it is important for future studies to investigate whether there is a convergence of pathways through which cytokines modulate lysosomal acidification to facilitate future therapeutic targeting [57].

## Metabolite and lipid dysregulation alter lysosomal pH

Dysfunctional microglial metabolism has been linked to neuroinflammation [58], as microglia play a key role in multiple cellular processes, including the production and response to signaling molecules [58]. In brains with neurodegenerative pathology, high levels of non-bioavailable iron in the form of ferritin are accumulated in the microglia, and lysosomal acidification has been shown to modulate iron content, thereby affecting microglial functions [59, 60]. On the other hand, there are other metabolites that can affect microglial lysosomal acidification and the associated phagocytic or autophagic functions, including glucose, myelin debris and lipids accumulation (Fig. 2c). Chronic exposure to high levels of glucose and lipids impair lysosomal function and alter microglial function [27, 61–63]. A study evaluating the effects of hyperglycaemia in astrocytes and primary rats microglia showed that chronic exposure to high glucose reduces mitochondrial membrane potential and ATP levels, which results in decreased lysosomal acidification and reduced degradation of Aβ oligomers [64].

Microglia actively participate in lipid metabolism, engaging in both lipid biogenesis and lipid catabolism processes [65]. On one hand, microglia produce essential lipids required for myelin formation and signaling within the brain [65]. On the other hand, microglia exhibit lipid catabolism capabilities through phagocytosis of cellular debris and myelin fragments [65], thereby playing an important role in the protective response to demyelination as they remove the resulting cellular waste, allowing neuronal regenerative processes to proceed [61]. It has been shown that an overload of myelin fragmentation results in the development of enlarged lysosomes and decreased lysosomal degradative function, contributing to microglia senescence and immune dysfunction in the normal aged mouse brain [27]. This suggests that increasing lysosomal acidification and hence microglial lipid catabolic activity is beneficial to myelin debris clearance. Interestingly, recent findings show that in case of severe demyelination in mouse, microglial lysosomal–autophagic pathway becomes overactivated, resulting in increased uptake of lipid into lysosomes, leading to lipid droplet accumulation and failed myelin debris clearance [66]. Lysosomal acidification inhibition with bafilomycin A1 treatment at the acute phase of demyelination is able to improve myelin debris degradation, indicating that an optimal lysosomal acidification in microglia is required to control the myelination process [66].

Triggering receptor expressed on myeloid cells 2 (TREM2) is a transmembrane protein essential for microglia proliferation and survival [67], and has been implicated in lipid imbalance and lysosomal dysfunction in microglia [68]. In an induced pluripotent stem cell (iPSC)-derived microglia (iMGLs) of Nasu–Hakola disease (NHD) with homozygous TREM2 mutations, dysfunctional lysosomal acidification and proteolytic abilities have been reported, together with impaired cholesterol metabolism, defective lipid droplet biogenesis, as well as altered lipid catabolic activity [68]. These changes were evident at the transcriptomic level in iMGLs, where genes implicated in cholesterol pathways (*CYP27A1, SOAT1, NCP2,* and *LPL*), lysosomal acidification (*ATP6AP2*), and autophagy (*LAMP2*) were downregulated in both iMGLs and NHD brain tissues. There is also a significant increase in genes associated with inflammation and immune activation in NHD patient brains, including inflammatory cytokines (*IL8, IL18, IL6R, TGFB1, IL17RA)* [68].

In a recent study, it was found that sleep deprivation increases microglial reactivity dependent on TREM2, and there is enhanced TREM2-dependent Aβ plaque deposition compared with mice with regular sleeping patterns [69]. In addition, sleep deprivation in 5xFAD mice with humanized TREM2 common variant led to reduction in lysosomal gene expression, such as *ATP6V0a1* and *CTSD*, indicating that sleep deprivation induced impairment of lysosomal degradation, potentially due to defective lysosomal acidification [69]. In another instance, TMEM106B deficiency has been shown to result in microglial lysosomal dysfunction and reduces the level of TREM2 in mice, leading to microglial dysfunction and myelination defects [70]. The progranulin protein, produced by the *GRN* gene, has been shown to be essential for maintaining lysosomal acidification in microglia [71]. In progranulin-deficient microglia, there is defective clearance and accumulation of myelin debris [72, 73]. These findings suggest that lipid imbalance and defective lysosomal acidification have a profound effect on modulating microglia inflammatory response and chronic neuroinflammation.

## ATP level and purinergic receptors signaling modulate lysosomal pH

P2X purinoceptors are trimeric ATP-gated cation channels formed by homomeric or heteromeric associations from seven different subunits (P2X1–P2X7). Among the seven P2X subunits, P2X4 receptor (P2X4R) and P2X7 receptor (P2X7R) signaling have been shown to modulate microglial function and mediating neuroinflammatory response [74–76]. The activation of P2X7R by ATP reduces lysosomal acidification in MG6 mouse microglial cells and primary microglia [75], resulting in increased lysosomal leakage and cytosolic lysosomal cathepsin B levels, and subsequently activating the NLR family pyrin domain containing 3 (NLRP3) inflammasome [19] (Fig. 2d). NLRP3 inflammasome activation can further lead to caspase-1 cleavage and IL-1β maturation and release from microglia, leading to the propagation of neuroinflammation and induction of neurotoxicity [19].

Within the P2X ion channel family, P2X4R has a unique subcellular distribution, being predominantly found on lysosomes while also being present on the plasma membrane [77]. P2X4R activation with the allosteric modulator ivermectin induces lysosomal acidification and potentiates myelin engulfment and degradation in experimental autoimmune encephalomyelitis (EAE) mouse model of multiple sclerosis (MS) [78]. On the other hand, the inhibition of the P2X4R by an antagonist TNP-ATP severely affected the removal and degradation of myelin debris by microglia, and enhanced the release of pro-inflammatory cytokines from microglia in EAE mice [78] (Fig. 2d). Interestingly, it has been shown P2X4R can be negatively regulated by lysosomal pH, where an acidic lysosomal pH inhibit P2X4R activity, while an elevated lysosomal pH in the presence of ATP activates the receptor [79].

Apart from playing an active role in modulating P2X purinoceptors signaling, ATP plays an important role in regulating lysosomal V-ATPase function, where increased ATP activity increases lysosomal acidification [14]. The soluble adenylyl cyclase (sAC) is an extensively expressed intracellular source of cyclic adenosine monophosphate (cAMP) as it catalyzes the conversion of ATP to cAMP and plays a key role in the colocalization of V-ATPase to lysosomes, hence leading to the modulation of lysosomal acidification [80]. Absence of sAC reduces cAMP levels and leads to lysosomal acidification impairments in primary mouse microglia [51]. These findings suggest the heterogenous nature of ATP and P2X purinoreceptors in modulating lysosomal acidification and function, and hence more studies are required to understand the intricate relationship between the different targets, to aid the development of specific therapeutic agents.

## Bidirectional relationship between toxic protein aggregates and lysosomal dysfunction

The accumulation of toxic protein aggregates such as tau and A*β* is associated with neurodegeneration [81–86] and microglia have been shown to play an important role in the internalization and degradation of these toxic protein aggregates [36, 87]. However, whether these protein aggregates are responsible for lysosomal deficits, or impaired lysosomal function arising from other insults results in the accumulation of toxic protein aggregates remains unclear. For instance, while some studies support the notion that the build-up of Aβ is a consequence of lysosomal dysfunction [55, 88], others show that Aβ impairs lysosomal functions and lead to deleterious downstream effects [26, 89, 90] (Fig. 2e).

Chronic exposure of BV2 mouse microglial cells to Aβ inhibited autophagic flux and induced lysosomal damage, shown by the presence of the acid hydrolase cathepsin-D in cytoplasm and increased LysoTracker staining in the cytoplasm indicative of lysosomal membrane permeabilization [91]. Prolonged Aβ exposure has also been observed to create an efflux of the nuclear TFEB, a regulator of lysosomal biogenesis, out from the nucleus, resulting in lysosomal acidification impairment and reduced Aβ clearance [26]. A small-molecule activator of TFEB, PF-11, when treated to primary rat microglia enhances TFEB nuclear translocation and mitigates the detrimental effect caused by soluble oligomeric Aβ on lysosomal function [90]. Aβ accumulation in microglia has also been shown to compromise PIEZO1 calcium signaling and reduce lysosomal activity [92]. PIEZO1 is an ion channel and mechanotransducer responsible for communication between the extracellular and intracellular environment. Restoration of PIEZO1 function by small-molecule agonist Yoda1 results in restoration of lysosomal activity and phagocytosis of Aβ [92]. Another type of intrinsically disordered protein, α-synuclein, has also been shown to disrupt microglial lysosomal acidification and inhibit phagocytic function in Parkinson’s disease (PD) [93].

While toxic proteins have been shown to result in lysosomal acidification defects, it has been suggested that toxic proteins can accumulate as a result of inherently weak acidification of microglial lysosomes. Compared with the lysosomes of macrophages and neurons, lysosomes of microglia are more weakly acidic, thereby limiting the function of lysosomal enzymes and hence their degradative activity [51, 90]. It was determined that the activities of PKA and ClC-7 chloride transporter, but not V-ATPase, are responsible for the pH difference between macrophage and microglial lysosomes [51, 55]. In addition, it was suggested that the ClC-7 is activated downstream of PKA, increasing lysosomal acidification in microglia [51]. Knockdown of ClC-7 in activated microglia was shown to lead to improper lysosomal acidification, resulting in impaired degradation of fibrillar Aβ [55]. In addition, it was also found that ClC-7 is mistargeted in quiescent microglia, leading to similar cellular and metabolic deficits. This is because delivery of ClC-7 to the lysosome is dependent on association with the OSTM1, the expression of which is decreased in resting microglia [55]. Interestingly, a study has shown that on the contrary, it is the acidic environment of endosome/lysosomal environment that can accelerate toxic Aβ oligomers formation when uptaken from the extracellular environment [94]. However, the same study also mentioned that the accumulation of Aβ oligomers can lead to further impairment of lysosomal function [94].

To monitor Aβ degradation in glial cells with respect to lysosomal pH, Aβ^pH^ was developed [95]. Aβ^pH^ is a human Aβ analog that possess similar aggregation characteristics as synthetic Aβ, but is non-fluorescent at physiological condition around pH 7.0–7.5 and fluorescent in mildly acidic environments of pH 4.5–5.0. Aβ^pH^ has been applied to show that phagocytosis of Aβ^pH^ was more prominent in microglia relative to astrocytes both in primary mouse microglial cell lines and in wild-type mouse [95]. In future studies, monitoring the rate of Aβ^pH^ uptake and clearance in microglia with different genetic alterations will allow for insights into the key players responsible for the lysosomal and phagocytic dysfunction in this pathway.

## Lysosome physiology and cathepsin activity

Lysosome size and positioning play a role in modulating lysosomal pH in CNS cells including microglia [96]. Enlarged lysosomes in CNS cells display reduced acidity and reduced lysosomal enzyme activity [97]. In microglia, TMEM106B overexpression has been shown to lead to accumulation of large lysosome-associated membrane proteins (LAMP) positive lysosomes, although the lysosomal pH was not quantified [98]. Interestingly, inhibition of lysosomal V-ATPase activity increases TMEM106B expression levels, strongly indicating the association between TMEM106B-induced lysosome enlargement and lysosomal acidification. In Gaucher disease, altered lysosomal localization or positioning has been shown to be early-onset of microglial activation and neuronal loss [99]. Using HeLa cells as a model, it has been shown that lysosomes located at the cell periphery are less acidic than juxtanuclear ones while maintaining similar luminal buffering capacity [96]. The reduced acidification capability of peripheral lysosomes is due to decreased V-ATPase activity and increased passive permeability to protons, thereby decreasing the overall proton concentration in lysosome lumen [96]. Future studies can be conducted to validate if similar regulatory mechanism is conserved in microglia.

**Fig. 3:**
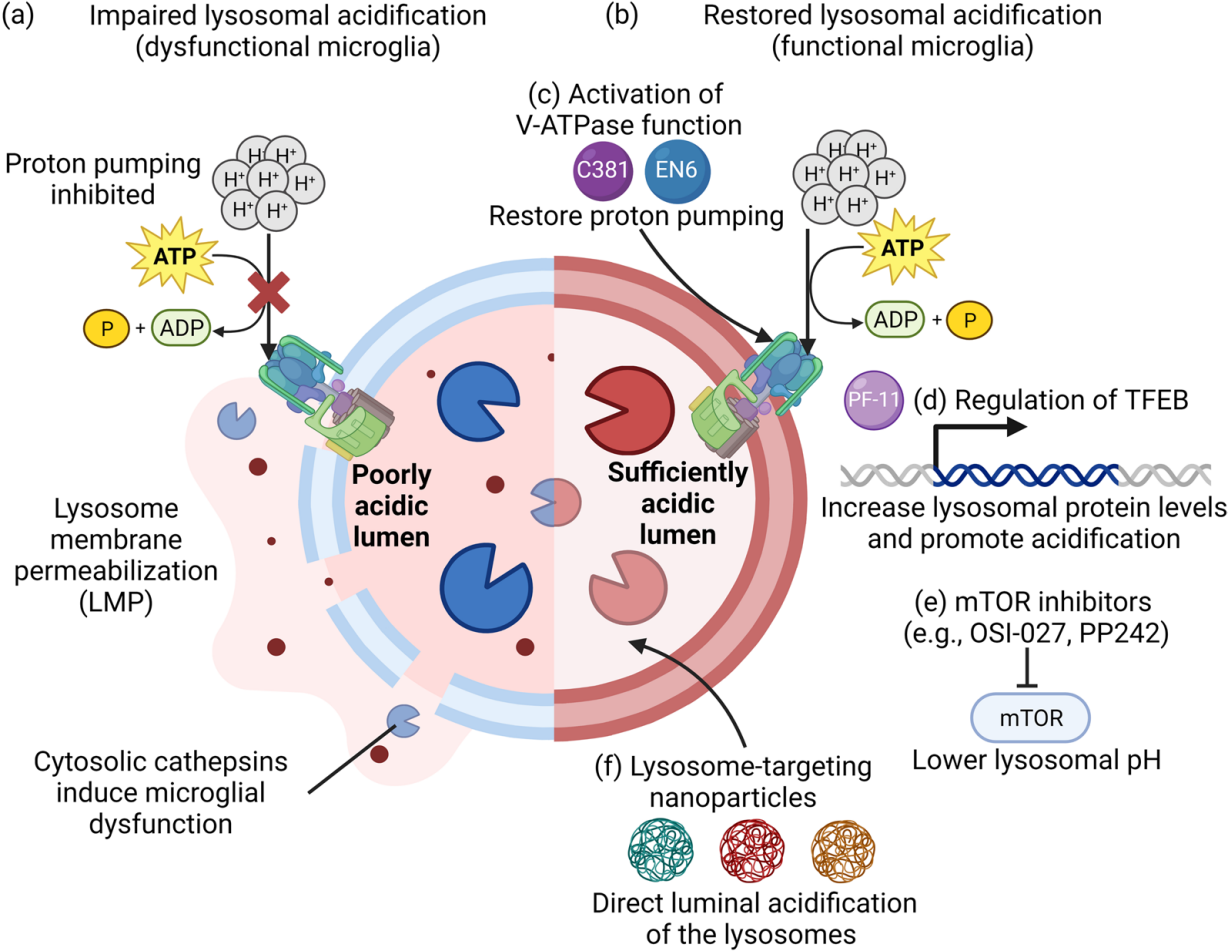
An overview of lysosome-targeting therapeutic agents developed to reacidify impaired lysosomes under diseased conditions. **a** Impaired lysosomal acidification results in lysosomal membrane permeabilization, leading to the release of cathepsins into cytosol that impair microglial function. **b** Lysosome reacidifying agents can restore lysosomal function to counteract undesired downstream consequences including LMP. Current lysosome-targeting therapeutic strategies have focused on using **c** small-molecule modulators such as C381 and EN6 to activate V-ATPase activity, **d** small-molecule activator of TFEB (PF-11) to increase lysosomal protein transcription and expression as well as promote lysosomal acidification, **e** mTOR inhibitors such as OSI-027 and PP242 to lower lysosomal pH, and **f** lysosome-targeting acidic nanoparticles to directly acidify the lysosome lumen. The figure was created with BioRender.com

Lysosomal membrane integrity is maintained by highly glycosylated transmembrane proteins, such as LAMP1 and LAMP2, as well as sphingolipids and cholesterol, which all serve to protect the membrane from degradation by lysosomal enzyme [100]. Maintaining lysosomal integrity is essential to preserve lysosomal function, including lysosomal acidification and enzyme activity, and is therefore crucial for cellular homeostasis. Different forms of cellular stress can induce LMP, resulting in the release of intralysosomal components such as cathepsins to the cytoplasm, inducing lysosomal-dependent cell death [91, 100] (Fig. 3a). Furthermore, cathepsins release can increase the release of inflammatory cytokines from microglia which trigger further neuroinflammation [101]. For instance, cathepsin C release has been shown to promote microglial neurotoxic polarization and aggravates neuroinflammation [101]. Cathepsin H could increase the release of IL-1β and interferon-gamma (IFN-γ) from microglial BV2 cells, as well as induce neuronal death [101]. Cathepsin X has been demonstrated to be associated with inflammation-induced neurodegeneration [102]. Maintenance of proper lysosomal and autophagic function through increasing lysosomal biogenesis via TFEB overexpression or by inducing autophagy with rapamycin have been shown to ameliorate LMP induced cell death [103, 104]. Hence, it is imperative for the development of therapeutic agents or strategies that can restore lysosomal acidification to counteract undesired downstream consequences due to LMP (Fig. 3b).

## Lysosomal pH monitoring strategies and therapeutic agents for reacidification

Fluorescent probes and reporter plasmids are common methods used for measuring lysosomal pH changes in microglia [105–107]. To increase the specificity to measure pH in the lysosomes organelle component, a more recent development is a tandem fusion of a pHluorin-mCherry linked to the luminal domain of the LAMP1 lysosomal marker protein, along with a cytosolic 3xFLAG tag that can efficiently isolate and purify lysosomes for evaluation of their function [108]. To detect lysosomal pH in vivo, the Nixon group has developed an autophagy reporter mouse expressing mRFP-eGFP-LC3 in neurons [37]. For future applications in lysosomal pH monitoring in microglia, the promoter can be changed from neuronal specific Thy1 promoter to TMEM119 or CXCR3 microglial specific promoter. Recent development of non-invasive lysosomal pH in vivo monitoring probe involve detection with near-infrared light, which only affords a penetration depth of a few millimeters into the tissues [109], making the application of this technique in CNS to be further investigated.

Given the wide-ranging cellular implications of lysosomal acidification in microglia, targeting and restoration of defective lysosomal acidification in microglia represents a promising therapeutic avenue for neurodegenerative diseases. Various therapeutic agents including small molecules that activate V-ATPase function (Fig. 3c), regulate TFEB expression (Fig. 3d), and inhibit mTOR signaling (Fig. 3e), as well as lysosome-targeting nanoparticles have been developed (Fig. 3f) [110, 111] and some have been applied for the restoration of impaired microglial lysosomal acidification. In particular, C381 and PF-11 have demonstrated capability to acidify microglial lysosomes [90, 112]. C381 has been reported to stimulate lysosomal acidification, mitigate lysosomal membrane permeabilization, and reduce neuroinflammation and neurodegeneration in mouse models of frontotemporal dementia and PD [112]. Compared to other cell types, it appears that C381 is significantly more effective in restoring lysosomal function in microglia, although whether there is any cell-specific effect remains to be illuminated [112]. By acting through alternative mechanisms, the small-molecule PF-11 has demonstrated the ability to enhance lysosomal acidification and function in microglia by increasing TFEB nuclear translocation and the expression of V-ATPase [90]. Lysosome-directed nanoparticles (NPs) are another type of therapeutic agent that has shown efficacy in improving lysosomal acidification [110, 113–118]. These NPs have been applied in both in vitro and in vivo models to restore lysosomal acidification in various cell types, including neuronal lysosomes, although their effects in modulating microglial lysosomal pH have not yet been characterized. Future screening studies can be conducted using cell-based assays with fluorescent probes and reporter plasmids, as well as artificial lysosomes based on polymersomes loaded with trypsin to discover novel modulators of lysosomal pH [119, 120].

## Conclusions

Microglial lysosomal acidification defects have been shown to result in inhibition of phagocytosis and autophagy, leading to the release of pro-inflammatory cytokines and the accumulation of toxic proteins, perpetuating neuroinflammation and exacerbating neurodegeneration. While the above discussion is based on stimuli-induced conditions, it remains important to investigate, under no inflammatory stimulation, whether early-onset genetic mutations or senescent microglia under normal aging would develop lysosomal acidification, and if these lysosomal acidification defects would initiate early neuroinflammation. In addition, while most of the studies adopted strategies to reacidify impaired lysosomes to maintain normal microglial function, some studies have observed that over-acidification of lysosomes can also lead to undesirable effects. Hence, this suggests that there is an optimal lysosomal pH level to be achieved for proper microglial function.

Previously thought to occupy a distinct cellular role, one organelle whose function has come to be recognized as highly intertwined with the lysosome is the mitochondria [97, 121], where the organelle that is damaged first is likely to encourage the subsequent impairment of the other, thereby establishing a complex lysosome–mitochondria crosstalk [59]. Defective lysosomal acidification can induce autophagic dysfunction which may further lead to defective turnover of mitochondria, which results in the accumulation of older mitochondria that generates significant amounts of reactive oxygen species in the microglia [122], which propagates inflammation [122]. On the other hand, mitochondria is the major producer of intracellular ATP, which is an important energy source used by the V-ATPase on lysosomes to maintain proper lysosomal acidification [121]. The contribution of impaired lysosome–mitochondria crosstalk to the pathology of AD, PD, and MS has been widely reported [121, 123, 124], although the mechanisms underlying these deficits remain somewhat elusive and more investigations are required to elucidate this bidirectional relationship [97].

To maintain homeostatic function in the brain, microglia cooperate with neurons and astrocytes, and their functions in turn affect microglia [54, 59]. It has been shown that neuronal damages affect lysosomal acidification in microglia [125]. Microglia and astrocytes both respond to neuronal injury with processes such as phagocytosis, proliferation, and morphological alterations [126–128]. Crosstalk between microglia and astrocytes is maintained in part via secreted factors including cytokines, chemokines, growth factors, neurotransmitters, as well as extracellular vesicles and tunneling nanotubes [129–131]. Hence, the role of microglial lysosomal acidification and function can be further complicated by the crosstalk between CNS cells, and further investigations are needed to clarify this complex relationship for improved understanding of the mechanisms underlying brain health and disease.

## Data Availability

Not applicable.

## Abbreviations

Aβ: β-Amyloid
AcNPs: Acidic nanoparticles
AD: Alzheimer’s disease
APP: Amyloid precursor protein
APP-βCTF: APP β-C-terminal fragment
ATP: Adenosine triphosphate
BBB: Blood–brain barrier
cAMP: Cyclic adenosine monophosphate
CNS: Central nervous system
EAE: Experimental autoimmune encephalomyelitis
FAD: Familial Alzheimer’s disease
IFN-γ: Interferon-gamma
iMGL: IPSC-derived microglia
iPSC: Induced pluripotent stem cells
LAMP: Lysosome-associated membrane proteins
LMP: Lysosomal membrane permeabilization
LPS: Lipopolysaccharide
MCSF: Macrophage colony-stimulating factor
NF-κB: Nuclear factor kappa B
NHD: Nasu–Hakola disease
NLRP3: NLR family pyrin domain containing 3
NP: Nanoparticle
OSTM1: Osteoporosis-associated transmembrane protein 1
P2X4R: P2X4 receptor
P2X7R: P2X7 receptor
PaNP: Photo-activated nanoparticle
PD: Parkinson’s disease
PKA: Protein kinase A
PKB: Protein kinase B
PLGA: Poly(lactic-co-glycolic acid)
PS1: Presenilin 1
PS2: Presenilin 2
sAC: Soluble adenylyl cyclase
SNARE: Soluble *N*-ethylmaleimide–sensitive factor attachment protein receptor
TFEB: Transcription factor EB
TREM2: Triggering receptor expressed on myeloid cells 2
UV: Ultraviolet
V-ATPase: Vacuolar (H+)-ATPase
